# Application and Progress of Combined Mesenchymal Stem Cell Transplantation in the Treatment of Ischemic Cardiomyopathy

**DOI:** 10.1155/2015/568502

**Published:** 2015-07-29

**Authors:** Ping Hua, Jian-Yang Liu, Jun Tao, Song-Ran Yang

**Affiliations:** ^1^Department of Cardiovascular Surgery, Sun Yat-sen Memorial Hospital, Sun Yat-sen University, Guangzhou 510120, China; ^2^Department of Experimental Psychology, University of Oxford, Oxford OX1 3UD, UK

## Abstract

Treatment of ischemic cardiomyopathy caused by myocardial infarction (MI) using mesenchymal stem cell (MSC) transplantation is a widely researched field, with promising clinical application. However, the low survival rate of transplanted cells has a severe impact on treatment outcome. Currently, research is focused on investigating the strategy of combining genetic engineering, tissue engineering materials, and drug/hypoxia preconditioning to improve ischemic cardiomyopathy treatment outcome using MSC transplantation treatment (MSCTT). This review discusses the application and progress of these techniques.

## 1. Introduction

Ischemic cardiomyopathy caused by myocardial infarction (MI) continues to be one of the major diseases that threaten human health. MI and ischemic cardiomyopathy lead to heart failure, could increase the socioeconomic burden, and increase the risk of death associated with these complications. Despite heart transplantation, little can be offered to patients suffering from end-stage heart failure, and this is where stem cell therapy stands up. Differentiation of transplanted stem cells into cardiomyocyte-like cells and the corresponding paracrine effect enables stem cell therapy (SCT) for ischemic cardiomyopathy to replenish the lost myocardium, reduce cardiomyocyte apoptosis in the infarction region, alleviate ventricular reconstruction, and improve short-/long-term cardiac function in patients. SCT overcomes many disadvantages of traditional therapies and has therefore become a research hot spot in the field of ischemic cardiomyopathy treatment [1].

Mesenchymal stem cells (MSCs) possess unique properties that may make them a better option for cardiac repair than other cell types. Unlike other adult stem cells, they appear to escape allorecognition by the immune system and they have immune-modulating properties, thus making it possible to consider them for use as an allogeneic cell therapy product. There is a large and growing body of preclinical and early clinical experience with MSC therapy that shows great promise in realizing the potential of stem cell therapy to effect repair of damaged cardiac tissue. MSCs are favored progenitor cells because they could produce a variety of beneficial paracrine cytokines, promote the generation of capillary and endogenous cardiomyocytes in the infarction region after transplantation, are abundant in source, and present low immunogenicity [2, 3]. Many randomized control trials (RCTs) [4–6] and corresponding meta-analyses [7, 8] have shown that transplantation of MSCs can safely and effectively increase left ventricular ejection fraction (LVEF), reduce the infarction area, and improve cardiac function. However, some RCTs [5] and meta-analyses [9] have shown that improvement of cardiac function resulting from MSC transplantation is limited.

One major limiting factor in MSC transplantation therapy (MSCTT) is the low survival rate of the transplanted progenitor cells. In fact, progenitor cell survival rate 4 days after transplantation is reported to be lower than 0.44% [10, 11]. This is thought to be due to local inflammatory reactions, ischemia, and hypoxia, production of cytotoxic cytokines from necrotic myocardial tissues, and lack of stromal survival signals/intercellular contacts [12]. The efficacy of MSCTT is closely related to this cellular survival rate [13]. To enhance survival of transplanted stem cells, Pouzet et al. [14] found that an increase in the number of transplanted cells improves the efficacy, and the results showed that “the dose of the injected cells was linearly related to the treatment efficacy.” Meluzín et al. [15] carried out the first clinical study to investigate the relationship between transplanted stem cell dose and treatment outcome in 2006. At the 3-month follow-up survey, they found that the magnitude of improvement in LVEF among patients was significantly dependent on the dose of transplanted cells. In contrast, other studies obtained opposing results. Studies by Strauer et al. [16] and Assmus et al. [17] showed that patients benefited equally when subjected to different doses of transplanted cells. Evidence-based TOPCARE-AMI clinical trial (clinical trial number: NCT00711542) [18] indicated that the dose was not correlated with improvements in left ventricular function and reduction of infarction area. Some studies have even reported that an increased number of cells might actually worsen the LVEF [16]. Some researchers suggested that the limited supporting substances for cellular survival within the cardiomyocyte microenvironment would only assure a certain number of cells to survive. Therefore, the higher the number of transplanted cells, the more difficult for cells in the middle of the cell mass to obtain enough oxygen and nutrients and the higher the rate of cell death [19]. Additionally, a higher number of transplanted cells might stimulate the cellular apoptosis process, which would fail to increase the corresponding cellular survival rate in the infarction region [20]. Because the relationship between cell dose and outcome of the MSCTT is still controversial, research is focused on improving the survival and treatment outcomes of MSCTT through multiple methods. These include application of genetically engineered MSCs, coupled with suitable tissue engineering materials, or preconditioning with pharmaceutical and ischemic substances.

## 2. MSCTT Combined with Genetic Engineering Technology

MSCs are not only ideal progenitor cells but are also good vectors. They can maintain stable expression of exogenous genes, even during the process of proliferation and differentiation. Genetic engineering introduces target genes into MSCs, increasing their survival rate and expression of specific proteins, thus improving their treatment outcome. In 2001, Lee et al. [21] reported that MSCs maintained stem cell activity and stability after a series of transfections of exogenous genes and also assured stable expression of those exogenous genes. Also D. Stewart reported the genetic modification of stem cells in the clinical trial The Enhanced Angiogenic Cell Therapy-Acute Myocardial Infarction Trial (ENACT-AMI, clinical trial number: NCT00936819) [22]. Consequently, transfection of specific genes into MSCs has been investigated and used to increase their survival capability, strengthen their antiapoptosis signaling pathway, or promote secretion of a variety of specific paracrine cytokines, to significantly and favorably enhance the outcome of MSCTT. More recently, however, it has become apparent that many of the functional improvements attributed to MSCs may be due to paracrine actions in the host tissue rather than cell differentiation and repopulation. A resultant shift in research has seen the emergence of studies aiming to elucidate the paracrine mechanisms underlying tissue repair and regeneration with MSCs transplantation.

Hypoxia is a major reason for the low survival rate of transplanted MSCs [23]. Within MSCs, hypoxia can activate the apoptosis protein Bax, lower the Bcl-2/Bax ratio, and alter mitochondrial permeability, leading to the release of cytochrome C and caspase-3 (CASP3). CASP3 inhibits expression of the oncogene Bcl-2, which eventually causes cell apoptosis. Bcl-2 is an important antiapoptotic gene capable of blocking the apoptosis signal transduction pathway, regulating mitochondrial function, and protecting the cell. Li et al. [24] introduced the Bcl-2 gene into MSCs and found that the optimized MSCs could better tolerate hypoxia in the infarction region. They also found that cellular survival rate, vascular density, and recovery of cardiac function in experimental animals were all improved. The CASP3 gene is a key effector protein during the process of cellular apoptosis [25]. Liu et al. [26] interfered with the CASP3 gene using siRNA in the MSCs and found that gene silencing significantly downregulated CASP3 expression and increased survival rate of the MSCs under the hypoxic environment.

The PI3K/AKT pathway is an important antiapoptotic pathway and plays roles in multiple biological processes, including cellular metabolism, the cell cycle, and cellular apoptosis. Studies have shown that activation of AKT can effectively protect the ischemic cardiac muscle [27]. Mangi et al. [28] showed for the first time that AKT-overexpressing MSCs injected into the infarction region of the rat heart survived well with decreased collagen deposition and inflammatory response and reduced infarction region/ventricular remodeling. Gnecchi et al. [29] also found that AKT-transfected MSCs had an increased secretory function and strong antiapoptotic capability; therefore, their survival rate after transplantation was significantly higher than the control group.

Heat shock proteins (HSPs) are a group of proteins induced by heat shock. Studies have shown that MSCs with high levels of HSPs have an increased survival rate. This may be related to the increased AKT activity and enhanced secretion of cytokines, including vascular endothelial growth factor (VEGF), fibroblast growth factor-2 (FGF-2), and insulin-like growth factor-1 (IGF-1), in the presence of HSPs. Chang et al. [30] and Wang et al. [31] modified MSCs with HSP70 and HSP20, respectively, and found that their capabilities for survival, differentiation, and proliferation were all improved. Matsumoto et al. [32] and Hua et al. [33] transfected MSCs with VEGF and found that the capillary density in the infarction region was significantly increased and that cardiac function was also significantly improved. In addition, Tang et al. [13] transfected MSCs with heme oxygenase 1 (HO-1) and transplanted the modified cells into the infarction region in mice to improve the treatment outcome via an immunoregulatory effect. The results showed that, on day 7, MSC survival number was increased 5-fold, ventricular remodeling was inhibited, and cardiac function was significantly improved, as compared to the control. Transfection of MSCs with either tumor necrosis factor receptor (TNFR) by Bao et al. [34] or CC-type chemokine receptor 1 (CCR1) by Huang et al. [35] resulted in decreased apoptosis.

In addition to studies of reduced MSC apoptosis following combined genetic engineering, introduction of genes that stimulate MSC differentiation has also been carried out. For instance, Zhou et al. transfected MSCs with HCN2 [36] and GATA-4 [37] prior to stimulating their differentiation into pacemaker-like cells. Other studies have tested multiple gene transfections to obtain a better outcome. Yau et al. [38] introduced both the VEGF and IGF-1 genes into MSCs and the results showed that this combined transfection could further reduce cellular apoptosis and improve cardiac function compared with individual transfections. This suggests synergism exists for multiple transfected genes. However, some research suggests that modification with two or more genes might greatly increase biological risks and lead to tumor formation [39].

From the perspective of transfection techniques, most studies have used a virus as the target gene vector [40]. Commonly used viruses include adenovirus, retrovirus, and lentivirus. Some studies have also applied nonviral delivery systems, such as liposomes, polymers, electroporation, and nuclei transfection [41]. Continuous advances of these techniques may further improve treatment outcome of their combined application with MSC transplantation in genetic engineering.

## 3. MSCTT Combined with Tissue Engineering Materials

Transplantation treatments which combine tissue engineering materials with MSCs provide MSCs with a scaffold for attachment and growth. Using this approach, cells can easily enter the defect site, carrying multiple cytokines/drugs to improve the surrounding environment of the infarction region, and stimulate survival/differentiation of the transplanted cells. However, some materials themselves can prevent or postpone ventricular remodeling by inhibition of collagen fiber hydrolysis within the cardiomyocytes and reduction of ventricular wall stress [42]. There are two major categories of cardiac tissue engineered materials: (1) the cardiac patch is seeded with MSCs to construct engineered heart tissue (EHT) and can be placed on the heart surface during surgery; (2) gel scaffolds made of gel materials can be used to encapsulate MSCs and be directly injected into the heart surface.

Collagen protein is often used as a primary material in the cardiac patch. Simpson et al. [43] seeded MSCs onto a cardiac patch made of collagen protein and found that over 90% of MSCs survived longer than 5 days. The same patch, which was subjected to* in vitro* culture with MSCs for 4 days, was placed on the myocardium in the infarction area. One week after transplantation, left ventricular fractional shortening was reduced by 30% in the treated animals compared with the control group. This suggests that application of the cardiac patch could effectively introduce MSCs into the myocardium in the infarction region and alleviate postinfarction ventricular remodeling. Chen et al. [44] used glycerol sebacate (PGS) to prepare the cardiac patch and found that stem cells on the patch could effectively transform into cardiomyocytes and maintain their activity and beating for over 3 months. The rat did not present any adverse reactions after the patch was stitched onto the left ventricle for 2 weeks. Similarly, Liu et al. [45] and Xiang et al. [46] prepared the cardiac patch using fiber protein and type I glycosaminoglycan, respectively. In both cases, a certain degree of improvement was observed for MSCTT after placing them onto the rat infarction regions.

Some studies have prepared patches with cytokines or drugs. Davis et al. [47] coupled IGF-1 to the scaffold fibers of the cardiac patch through biotinylation. The controlled release of IGF-1 activated AKT to promote survival of the transplanted cells. This method could increase survival of the transplanted cells and prevent the concern of tumor proliferation resulting from transgenesis. Perets et al. [48] prepared porous alginate scaffolds incorporating poly(lactic-co-glycolic acid) microspheres capable of controlling the release of angiogenic factors and successfully induced proliferation of the transplanted cells. Dvir et al. [49] applied an even more unique method to incorporate cells, growth factors, and angiogenic factors into an alginate scaffold, which was used to construct the cardiac patch prior to transplantation into the rat omentum. Because the omentum is rich in blood supply and lymphocytes secret multiple cytokines, this significantly promoted vascularization of this patch. Seven days later, the patch was explanted and then transplanted onto the rat heart surface. Twenty-eight days after transplantation, the vascularized patch was better integrated into the rat heart, ventricular remodeling was inhibited, and heart function was improved.

Aside from the cardiac patch technology, some gel-like alginates, cellular matrices, and fiber proteins have been constructed into injectable materials and injected in combination with MSCs into the heart surface. This method avoids invasive open surgery and has other advantages. In studies investigating the combined application of cell transplantation and gel materials, Christman et al. [50, 51] confirmed for the first time in experimental animals that injection of fibrin gel containing skeletal myoblasts into the left ventricle of the rat could boost the cell survival rate, reduce the infarction area, increase vascular density, and effectively improve myocardial ischemia, compared with injection of fibrin alone. Many subsequent studies support the use of combined stem cells and liquid gel materials for further improvement in cell transplantation therapy outcome [52, 53].

Combining tissue engineering strategies with MSCTT has been applied in multiple clinical trials [54, 55]. The results have shown that this combination is safe and feasible. However, larger-scale RCTs are still needed to assure the long-term safety and effectiveness of this combination protocol.

## 4. Drug/Hypoxia Preconditioning

The biological properties of clinical drugs allow them to precondition MSCs or patients who are about to receive cell transplantation to improve MSCTT outcomes through multiple mechanisms. Yang et al. [56] pretreated experimental animals with atorvastatin, 3 days prior to MSC transplantation, and found this drug could improve the cardiac microenvironment and promote the survival rate of transplanted MSCs by increasing myocardial perfusion, suppressing cardiomyocyte apoptosis, reducing oxidative stress, and inhibiting inflammatory reactions. An* in vitro* hypoxic model later indicated that atorvastatin mainly inhibited MSC apoptosis through the activation of endothelial nitric oxide synthase, mediated by the PKA pathway [57]. Zhang et al. [58] directly preconditioned MSCs with atorvastatin and found that it could protect the transplanted MSCs under hypoxia through activation of the MSC autophagy to significantly improve the survival rate. Studies have also found that the traditional Chinese medicine, Huangliansu, can inhibit hypoxia-mediated cell apoptosis* in vitro* and suggest that the mechanism might be related to inhibition of the mitochondrial apoptotic pathway mediated by Zhang et al. [59]. Additionally, many other drugs such as diazoxide, captopril, nicorandil, minoxidil, bimakalim, and hydrocortisone also improve the survival rate of stem cells.

Hypoxic preconditioning refers to the hypoxic culture of MSCs before transplantation. In 2006, Xie et al. [60] reported for the first time that MSCs subjected to hypoxic preconditioning could differentiate into cardiomyocyte-like cells. Current studies suggested that hypoxic preconditioning can activate the PI3K/AKT pathway and upregulate the expression of hypoxia inducible factor-1*α* (HIF-1*α*), to increase the antiapoptotic capability of MSCs [11]. Other studies have confirmed that hypoxic preconditioning also upregulated gene expression of the potassium channel Kv2.1 and activates focal adhesion kinase (FAK) to enhance migration and homing [61]. Hypoxic preconditioning is simple and produces many biological effects. Therefore, it is currently a common preconditioning method used before MSCs are transplanted into the infarction region. However, the optimal oxygen levels and cell culture time warrant further investigation.

## 5. Summary

Genetic engineering, tissue engineering materials, and drug/ischemic preconditioning could be applied individually or in combination during MSCTT. For instance, Ye et al. [62] introduced VEGF genes into cells more effectively using PEI nanoparticles. Drugs coupled to gels that can be controlled by enzymes or temperature could be more accurately released at specific times and locations. The combined application of a variety of these methods could further improve MSCTT outcomes by maximizing their individual strengths. MSCTT combined with these methods brings more ideal treatment outcomes and more study directions. In the future, this approach will contribute to improving and expanding current applications of stem cell treatments.
